# *Plasmodium vivax* aldolase-specific monoclonal antibodies and its application in clinical diagnosis of malaria infections in China

**DOI:** 10.1186/1475-2875-12-199

**Published:** 2013-06-12

**Authors:** Emmanuel E Dzakah, Keren Kang, Chao Ni, Hong Wang, Peidian Wu, Shixing Tang, Jihua Wang, Jufang Wang, Xiaoning Wang

**Affiliations:** 1 School of Bioscience and Bioengineering, South China University of Technology, University City, Panyu District, Guangzhou 510006, China; 2 National Engineering Laboratory of Rapid Diagnostic Tests, Guangzhou Wondfo Biotech Co., Ltd, Science City, Lizhishan Rd. No. 8Luogang District, Guangzhou 510663, China; 3 Institute of Life Science, General Hospital of Chinese People’s Liberation Army, Beijing 100853, China

**Keywords:** *Plasmodium vivax*, Aldolase, Malaria, Monoclonal antibodies, ELISA

## Abstract

**Background:**

Most rapid diagnostic tests (RDTs) currently used for malaria diagnosis cannot distinguish the various *Plasmodium* infections. The development of a *Plasmodium vivax* specific RDTs with high sensitivity to sufficiently differentiate the two most common *Plasmodium* infections would be very crucial for disease treatment and control.

**Method:**

*Plasmodium vivax* aldolase gene (PvALDO) was amplified from the extracted genomic DNA and constructed into pET30a vector. *Plasmodium vivax* aldolase protein was successfully expressed in *Escherichia coli* in soluble form and the overall purity was over 95% after one-step affinity chromatography purification. The purified products were used for the immunization of mice and rabbits. Rabbit polyclonal antibodies generated were deployed to develop a novel antibody-capture ELISA for hybridoma screening.

**Results:**

Three PvALDO specific mAbs (14C7, 15F1 and 5H7) with high affinities were selected and used in immunochromatographic test strips. Clinical blood samples (n=190) collected from Yunnan (China) were used for evaluation and the RDT’s sensitivity for *P. vivax* was 98.33% (95% Confidence Interval (CI): 91.03% to 99.72%) compared with microscopic examination. There was specificity of 99.23% (95% CI: 95.77% to 99.87%) for *P. vivax*. Only one *Plasmodium falciparum* sample was detected among the *P. falciparum* samples (n=20). All *Plasmodium malariae* samples (n=2) as well as healthy uninfected samples (n=108) were negative. Overall performance of this RDT was excellent with positive predictive value (PPV) and negative predictive value (NPV) of 98.33% and 99.23%, respectively, at 95% CI and a very good correlation with microscopic observations (kappa value, K=0.9757). Test strips show high sensitivity even at 6.25 ng/ml of recombinant *P. vivax* aldolase (rPvALDO).

**Conclusion:**

This study further elucidates the possibility of developing aldolase-specific RDTs which can differentiate the different *Plasmodium* infections and improve accurate diagnosis of malaria. This RDT could adequately differentiate between *P. vivax* and *P. falciparum* infections. The novel mAb screening method developed here could find application in the screening of highly specific antibodies against other antigens.

## Background

*Plasmodium vivax*, a causative agent of relapsing benign tertian malaria, is the second most important malaria causing species and afflicts several hundred million people every year [1,2]. The occurrence rate of *P. vivax* infection ranges between 70-90% in most of Asia and South America, 50-60% in South Eastern Asia and Western Pacific, and 1-10% in Africa [3].

Rapid and effective diagnosis of the disease is essential for combating and eradicating malaria in the world. Microscopic examination of thick and thin blood smears from patients have served as the gold standard in diagnosing malaria over the years [4]. However, the greater section of those patients affected by malaria reside in villages and the very remotes areas of the world, making trained personnel, microscopes and other equipment difficult to access. These shortcomings have necessitated the emergence of rapid diagnostic tests (RDTs). These tests are effective, quick and easy to use after short appropriate training. Until now, many RDTs have been developed with great focus on the detection of histidine-rich protein 2 (HRP-2) from *Plasmodium falciparum* and parasite specific lactate dehydrogenase (pLDH) or *Plasmodium* aldolase from all species [4]. Nevertheless, most of these RDTs have reported undesirably low sensitivities for the diagnosis of *P. vivax*[5] and are unable to correctly distinguish between *P. vivax, P. falciparum* or mixed infections, a situation that has adverse effects on the accurate treatment of the disease.

Aldolase is a major enzyme involved in the glycolytic cycle of *Plasmodium* and is released into the blood during infection or can be localized in the cytoplasm of the parasite in soluble forms [6]**]**. *Plasmodium falciparum* and *P. vivax* possess only one aldolase isoenzyme [5,7], and a high proportion of the amino acid sequences are relatively conserved in all *Plasmodium* species [8,9], making aldolase a pan-specific target antigen for *Plasmodium* detection. The *P. vivax* aldolase gene has 1100 base pairs which are translated into 369 amino acids with a molecular weight of 41KDa.

In this paper, the cloning and expression of soluble *P. vivax* aldolase proteins in *Escherichia coli* and the development of novel monoclonal antibody (mAb) screening methods for selecting high affinity antibodies is described. MAbs specific to the *P. vivax* aldolase were utilized in immunochromatographic tests for screening of clinical blood samples. This opens the door to the production of *P. vivax* specific RDTs that target the aldolase antigen and can therefore used as an alternative to augment the already existing malaria RDTs.

## Methods

### Materials

Restriction endonucleases, NdeI and SalI, and PrimeStar Hs mix, and PCR mix, DNA markers were purchased from TaKaRa Biotech Company (Dalian, China). pET-30a plasmid, competent *E. coli* cells (DH5α and BL21 (DE3)) and SP2/0-Ag14 myeloma cells were preserved in School of Bioscience and Bioengineering, South China University of Technology (China). RPMI 1640 medium, fetal bovine serum (FBS), kanamycin, penicillin-streptomycin, hypoxanthine and thymidine (HT), hypoxanthine-aminopterin-thymidine medium (HAT) and polyethylene glycol (PEG) were purchased from Gibco (California, U.S.A). Methyl cellulose was purchased from Sigma-Aldrich (St. Louis, U.S.A). All chemicals used were of higher molecular grade.

### Samples collection and examination

Field and clinical blood samples of *P. vivax* (n=60) and *P. falciparum* (n=20) from infected persons and normal blood from healthy uninfected individuals (n=108) were collected from the Yunnan Province, China. *Plasmodium malariae* samples (n=2) were supplied by the Yunnan Institute of Parasitic Research, China. All samples were read by two experienced microscopists. Giemsa-stained thick blood smears were examined by light microscopy for 100 thick-film fields. Parasite species and density in positive films were identified and recorded as the number of parasites per 200 white blood cells.

### PCR cloning and recombinant protein expression

Total genomic DNA was extracted from infected blood using Animal Genomic DNA Mini Preparation Extraction Kit purchased from New Probe Biotech Company (Beijing, China). A pair of oligonucleotide primers were designed, complementary to the forward and reverse strands of the *Pv*ALDO (aldolase) gene based on the putative sequence of *P. vivax* isolate KPValdo06-43 obtained from GenBank (Accession number: HQ230241.1). The forward primer, PV-ALD-F, '5-GACT**CATATG**GCCACTGGATCCGAATA-'3, and the reverse primer, PV-ALD-R, '5-TACA**GTCGAC**ATAGACGTACTTCTTTTCGTAAG-'3 were designed to contain the NdeI and SalI restriction sites, respectively. Electrophoresis of the PCR product on a 1% agarose gel gave a DNA band of the expected size which was excised and purified. The purified PCR product and the pET30a vector were double digested with NdeI and SalI restriction enzymes and cloned into pET30a vector.

The pET-30a plasmid containing the *Pv*ALDO gene was transformed into CaCl_2_ competent *E. coli* BL21 (DE3) cells. The transformed cells were verified by colony PCR. For small scale protein expression, starter cultures were grown overnight at of 37°C in 5 ml LB medium, supplemented with 50 mgml^−1^ kanamycin. About 100 μl of saturated overnight cultures were used to inoculate 10 ml cultures and then allowed to grow to OD 600 between 0.6 - 0.8 at 37°C before protein induction with 1 mM IPTG. Cultures were incubated at different temperatures of 30°C, in a shaker with and without induction with IPTG for 4 h. The cells were harvested by centrifugation at 6,000×g for 10 min. The cell supernatant was removed and the cell pellet was re suspended in lysis buffer consisting of 100 mM Tris–HCl, pH 8.8. Cell suspensions were disintegrated ultrasonically on ice and centrifuged at 6,000×g for 15 min. The protein content of the supernatant and pellet fractions were visualized on 12% SDS PAGE. Recombinant *P. vivax* aldolase (rPvALDO) protein was purified on a HiTrap Ni^2+^ column using AKTA Purifier (GE Healthcare, UK). The resulting pure protein (>95% purity) was buffer exchanged and stored at −20°C until immunization.

### Western blot analysis

SDS-PAGE was carried out on 12% separating and 5% stacking gels using the Laemmli discontinuous buffer system [10]. Samples were boiled for 5 min with SDS loading buffer prior to loading and then electrophoresed. Proteins were transferred to a polyvinylidene fluoride (PVDF) membrane in transfer buffer (25 mM Tris, 186 mM glycine, and 20% methanol). After blocking with 5% non-fat milk in 1X Tris-buffered saline (TBS) supplemented with 0.1% Tween-20, pH 7.4 for 2 h at room temperature, the membrane was then incubated in 1:2,000 anti-6X His tag antibody (Abcam, Cambridge, UK) for 2 h at room temperature. Membranes were washed in three changes of TBS-T. PvALDO proteins were visualized with Supersignal West Pico chemiluminescent substrate kit (Thermo Fisher Scientific Inc., Rockford, USA) and scanned with MicroChemi Western Blot Analyser (DNR Bio-Imaging Systems Ltd, Israel).

### Monoclonal and polyclonal antibody production

#### Animal immunization

Six-to-eight-week-old Balb/c mice purchased from Guangdong Animal Laboratory Center (Guangzhou, China), were injected subcutaneously with 100 μg of the purified rPvALDO protein in equal portion of complete Freund’s adjuvant (CFA), (Sigma, St. Louis, U.S.A), for the initial immunization. Three booster immunizations were done at two-week intervals with 50 μg protein in incomplete Freund’s adjuvant (IFA). The mice received a final booster injection with 100 μg protein intraperitoneally three days prior to cell fusion. A three-month old female rabbit of 2.5 kg was immunized with 1ml of rPvALDO-saline-adjuvant mixture containing 800 μg antigen for the initial immunization and 500 μg in three subsequent boosters at two-week intervals. This research does not violate any national guidelines and institutional policies for use of animals in research.

#### Polyclonal antibody purification and conjugation by periodate method

Blood (10 ml) was collected from the central vein of the rabbit and allowed to clot at room temperature for 2 h, incubated at 4°C for 2 h and then centrifuged at 6,000×g for 15 mins. Serum was purified by caprylic acid and ammonium sulfate precipitation. The purified rabbit anti-aldolase IgG (1mg) was labelled with horseradish peroxidase (HRP) by oxidation method [11]. The produced conjugates were tested by indirect ELISA method.

#### Hybridoma production and mAbs generation

Mice were sacrificed and the spleen cells were fused with the myeloma cell line SP2/0-Ag14 at a ratio of 10:1 using PEG 1500 as described by Kohler and Milstein [12]. The fused cells were then mixed with methylcellulose-RPMI 1640 media supplemented with 15% (v/v) FBS, 100 U/mL penicillin, 100 μg/mL streptomycin, 1% (v/v) HEPES, 2% (w/v) methyl cellulose, 1% (v/v) HAT) (Gibco, California, U.S.A), and plated on petri dishes (35mm). The dishes were incubated at 37°C under a 5% CO_2_ overlay for 5–7 days in humidified chambers. Fast growing hybridoma clones were picked into 96-well plates containing complete RPMI 1640 medium supplemented with 1% HT. Cell culture supernatants were screened using both indirect and a novel antibody-capture ELISA described below.

#### Enzyme-linked immunosorbent assays

Both the indirect ELISA assay and a novel antibody-capture ELISA screening methods were used in the selection of hybridomas that produce antibodies against the rPvALDO antigen. In the indirect method, the wells of microtitre plates were coated with 2 μg/ml of rPvALDO antigen in coating buffer (0.05 M carbonate buffer, pH 9.6) for 3 h. The plates were washed (0.015 M phosphate buffered saline (PBS) with 0.1% Tween 20) and blocked with 3% BSA for 2 h before the addition of 100 μl of cell culture supernatant for 1 h at 37°C. HRP conjugated goat anti-mouse IgG (ZSGB-BIO, Beijing, China) at 1:20,000 dilution was added to each well and incubated for 30 mins. The plates were then washed and enzymatic reaction was visualized using substrate TMB with hydrogen peroxide and stopped with 2 M sulphuric acid. Optical density was measured by spectrophotometer at wavelength of 450 nm.

In the novel antibody-capture ELISA method, the microtitre plates were coated with goat-anti-mouse IgG (GAM) at 2 μg/ml and incubated at 4°C overnight. After rinsing, the plates were blocked with 3% BSA for 2 hr. 100 μl cell supernatant was added to each well and incubated for 1 hr. Plates were washed and the rPvALDO antigen (2 μg/ml) was added and incubated for another 30 mins. After rinsing, 100 μl rabbit-anti-PvALDO IgG-HRP (1:5,000) diluted in 1% BSA containing 1% healthy mouse serum was dispensed into each well and incubated for 30 mins at 37°C and then rinsed. Enzymatic reaction was visualized as described above.

#### Ascites production and purification

Two adult F1 hybrid mice (Animal Laboratory, Sun-Yat-Sen University, Guangzhou, China) were injected intraperitoneally with mineral oil (0.5 ml/mouse) followed by injection with 5×10^6^ of hybridoma cells on the seventh day. Ten days later, ascites were collected and centrifuged at 6,000×g for 10 min. The IgG fractions were prepared by ammonium sulfate precipitation followed by purification on Protein A column. The titer of both ascites and purified mAbs were determined by antibody-capture ELISA.

#### Isotyping and affinity of mAbs

Commercial ISO-2 kit (Sigma, St. Louis, USA) was used in the determination of the isotypes of the monoclonal antibodies. Antibody affinity was determined and calculated as described by Raghava and Agrewala with little modifications [13]. Polystyrene 96-well plates were coated with GAM and serial dilutions (1.25 μg/ml to 0) of mAbs were added and incubated for 1 h. Serially diluted antigen concentrations (0.50, 0.25, 0.125, 0.0625 μg/ml) were then added for 30 mins. After rinsing, rabbit anti-ALDO IgG-HRP (1:20,000) was added and incubated for 30 mins at 37°C. Colour development was measured at 450 nm on a microtitre plate reader.

### Immunochromatographic test strips for the evaluation of clinical samples

Monoclonal antibodies 15F1 and 5H7 were used as capture and labelled antibodies respectively in an immunochromatographic assay. The 5H7 antibody was conjugated to colloidal gold and sprayed on glass fiber at 12 μl cm^-2^. Monoclonal antibody 15F1 (2.0 mg/ml) and goat-anti-mouse IgG (1.0 mg/ml) were sprayed on a nitrocellulose membrane at 1.5 and 1 μl cm^–1^ to form the test and control lines respectively. The glass fiber pad and the membrane were left to dry at 30°C overnight. During the assembly of the test kit, 0.6 cm of the colloidal gold labelled mAb 5H7 glass fiber was placed a few centimeters away from the mAb 5F1 test line with an absorption pad placed at the opposite end. The assembly was cut into 3.0 mm wide strips. A maximum of 5 μl blood was applied to the sample application site and a sample dilution buffer was added and the colour development observed for 15 mins. A red band in the test and control line zones is formed for positive samples and the absence of a band in the test line zone is indicative of negative results while test samples without the appearance of the controls are regarded invalid and, therefore, repeated. False positive or negative results are cross examined by PCR.

### Test strip sensitivity to recombinant aldolase

Serial dilutions of rPvALDO (100 ng/ml, 50 ng ml, 25 ng/ml, 12.5 ng/ml, 6.25 ng/ml, 3.125 ng/ml, 1.56 ng/ml, and 0.78 ng/ml) were applied to the test strips to check its sensitivity level at different antigen concentrations. Strips were observed for a maximum of 15 mins and the colour development was compared with a standard colour chart and the band intensity graded.

### Data analysis

The sensitivity and specificity of the immunochromatographic assay for the detection of *P. vivax* were compared with thick blood microscopic examination results by Kappa statistical analysis, K [14]. P<0.005 was considered as significant.

## Results

### Cloning and expression of recombinant protein

**Figure 1:**
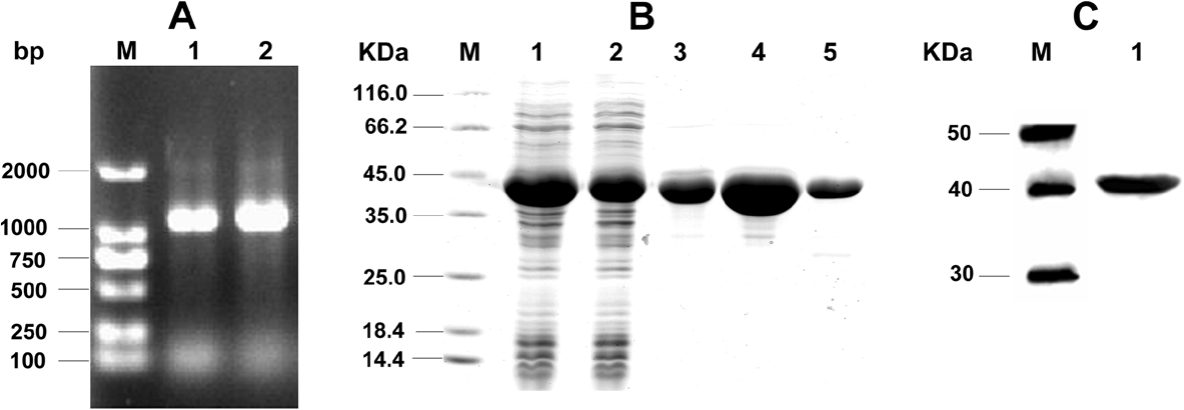
**Gene amplification and protein expression.** (**A**) PCR of PvALDO gene. M: DL2000 marker, 1 and 2 represent annealing temperatures 50.4°C and 52.4°C, respectively. (**B**) Protein expression and purification. M: Marker, lanes 1 and 2 are the whole cell lysate and supernatant after induction, respectively. Lanes 3, 4 and 5 are pooled portions of purified products. (**C**) Western blot analysis of PvALDO protein. M: Marker, 1: Purified PvALDO protein (10 μg).

The total *Plasmodium* genomic DNA from *P. vivax* infected blood was extracted with a concentration of 16.2 μg/ml. Two oligo primers were designed and modified to contain the NdeI and SalI restriction sites. Amplification of the full-length PvALDO gene was carried out and the expected DNA band size of 1.1 Kb was observed (Figure 1A). The PCR product and pET30a vector plasmid were digested with NdeI and SalI restriction enzymes, purified and ligated together. Ligation products were used to successfully transform competent BL21 (DE3) *E. coli* cells. Analysis of the cell pellets and supernatant by SDS PAGE after induction revealed over-production of the target protein with molecular weight of 41 kDa in the pellet for cells cultured at 37°C. Reducing the incubation temperature to 30°C showed marked expression of soluble proteins in the supernatant (Figure 1B). It was however observed that higher incubation temperatures result in the formation of inclusion bodies. Expression was then carried out at 30°C and purified on a HiTrap Ni^2+^ column with a total a purity of >95% (Figure 1B). Western blot analysis of the protein using rabbit-anti-His antibodies revealed the intact integrity of the protein (Figure 1C).

### Animal immunization

The sera of immunized mice showed high antibody titers after the second booster immunization. Relatively good titers were observed at 10^-5^ dilutions. Purified rabbit sera also showed very high antibody titers after the third booster immunization with titers still high at 10^-6^ dilution.

### Antibody titer and affinity

**Figure 2:**
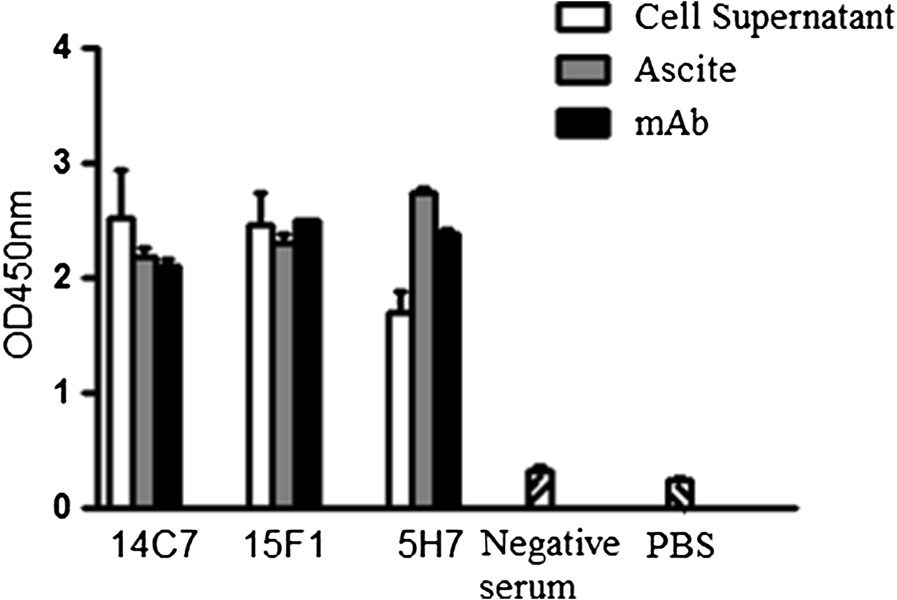
**Antibody titer of cell supernatants, ascites and purified antibodies.** The OD values (mean ± SD) of hybridoma cell supernatants, ascites (10^-3^ dilution), and mAbs (1 μg/ml) were measured by the antibody-capture ELISA at 450 nm. Polystyrene 96-well plates were coated with GAM (2 μg/ml), antibodies were added and rPvALDO used was at a concentration of 2 μg/ml and HRP conjugated rabbit-anti-ALD IgG (1:5000) was used as secondary antibody. PBS and normal mouse serum were used as control.

**Figure 3:**
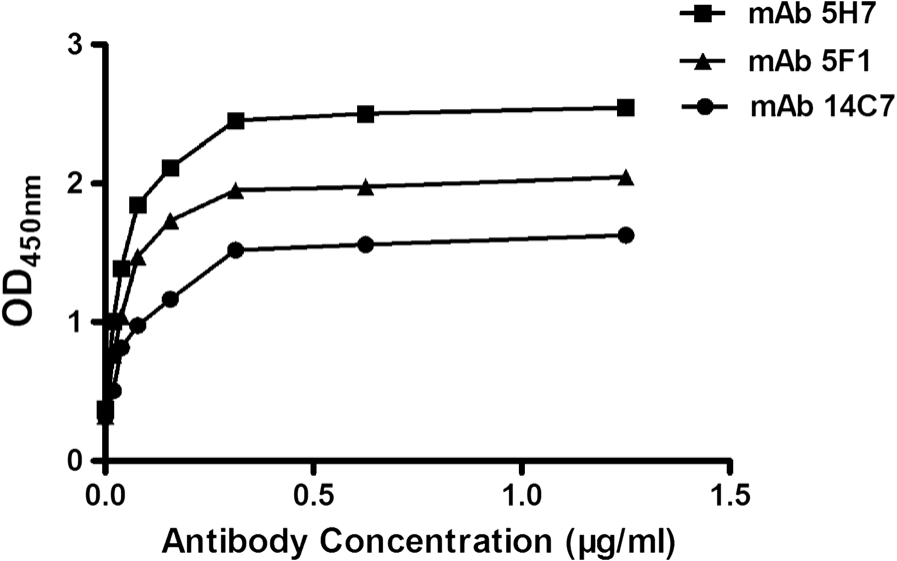
**Antibody affinity.** Plates were coated with GAM (2 μg/ml) and mAbs 14C7, 15F1 and 5H7 at concentrations of 1.25, 0.63, 0.313, 0.156, 0.078, 0.039 and 0.01953 μg/ml were added for 1h. rPvALDO (0.5 μg/ml) was added and PBST was used as negative control. Rabbit-anti-ALD IgG-HRP (1:5000) was used as secondary antibody.

The antibody titers of the three selected mAbs (14C7, 15F1 and 5H7) were high in cell culture medium, ascite and after Protein A purification (Figure 2). The affinity of these mAbs for rPvALDO was also determined at different antigen and antibody concentrations. Computed affinities were 1.69×10^6^, 2.36×10^7^, and 2.24×10^6^ M^-1^ for 14C7, 15F1 and 5H7, respectively (Figure 3).

### Comparison between indirect and novel antibody capture ELISA

The novel antibody capture ELISA proves to be more reliable in the screening of hybridoma clones. Clone 15F1 which was undetected by indirect ELISA due to extremely low optical density was easily identified using this method of screening (Table 1).

**Table 1:** Comparison between indirect and novel Ab capture methods

**mAb**	**Antibody screening method**	
	Indirect ELISA	Novel antibody capture ELISA
5H7	1.921	2.273
14C7	1.544	1.475
15F1	0.28	1.907
Negative control	0.09	0.105

### Characteristics of anti-PvALDO mAbs

Commercial ISO-2 kit was used in the determination of the isotypes of the mAbs. The isotype, purity and concentration of the purified antibodies are presented in Table 2.

**Table 2:** Characteristics of anti-PvALDO mAbs

**mAb**	**Isotype**	**Purity (%)**	**Concentration (mg/ml)**
5H7	IgG1	90	4.02
14C7	IgG1	85	2.56
15F1	IgG1	80	3.30

### Test strip sensitivity to recombinant aldolase antigen

**Figure 4:**
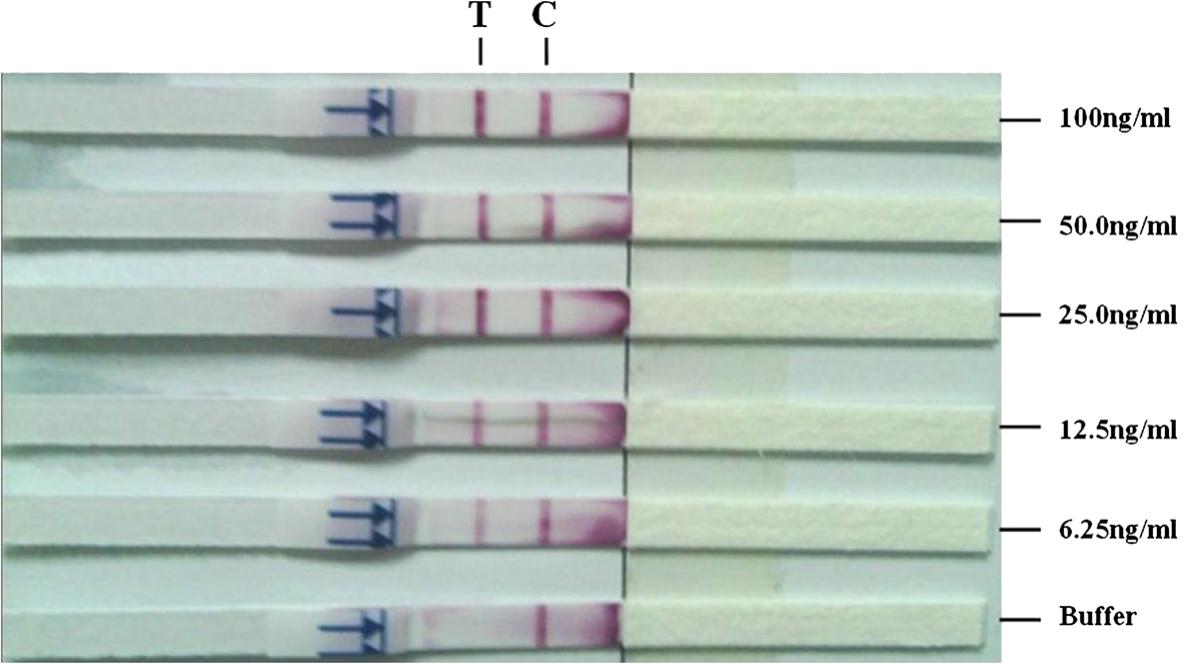
**Sensitivity of assay to rPvALDO antigen.** Test strips were treated with different concentrations of rPvALDO antigen and the colour development observed after 15 mins. T and C represent the test and control lines, respectively.

Different combinations of mAbs 14C7, 15F1 and 5H7 as either capture or detector antibody showed that all three mAbs could be used for the establishment of immunochromatographic assays. However, the 15F1-5H7 format, that showed the best sensitivity, was selected for further experimentation. The sensitivity of the test strips were determined by treatment with different antigen concentrations (100 ng/ml, 50 ng/ml, 25 ng/ml, 12.5 ng/ml, 6.25 ng/ml, 3.12 ng/ml, 1.56 ng/ml, and 0.78 ng/ml) and the band intensity was observed after 15 mins and compared with standard colour chart. Good assay sensitivity was observed up to 6.25 ng/ml of antigen (Figure 4).

### Comparison of immunochromatographic test strips and standard method

Test strips were used to evaluate *P. vivax* positive samples (n=60), of which 59 positives (59/60) and one false negative (1/60) was detected. Negative results were observed in all non-*P. vivax* samples (129/130), except for one *P. falciparum* sample that was positive for both test strip and PCR cross examination. The parasitaemia density of this sample was 4,560 parasites/μl and even at this density, the positive line was extremely faint and hardly visible to the naked eye. All serial dilutions testing of this *P. falciparum* sample were negative. All *P. malariae* samples (n=2) were negative (Table 3). Agreement between the immunochromatographic test strips and thick blood smear reference method for the 190 whole blood samples were analysed (Table 4). The specificity and sensitivity of these test strips, compared with microscopic examination were 99.23% (95% Confidence Interval (CI): 95.77% to 99.87%) and 98.33% 95% CI: 91.03% to 99.72%, respectively. Positive predictive value (PPV) and negative predictive value (NPV) of 98.33% and 99.23% respectively were also observed at 95% confidence interval. There was also a strong agreement (K = 0.9757) between the immunochromatographic test strips and the standard method.

**Table 3:** Band intensities of immunochromatographic test strips.

	Detection level*					
***Plasmodium spp*. (density)**	**Negative**	**Faint**	**Weak**	**Medium**	**Strong**	**Total**
*P. vivax* (1-499/μl)	1	9	5			**15**
*P. vivax* (500-1000 μl)			7	10		**17**
*P. vivax* (>1000 μl)			5	18	5	**28**
*P. falciparum*	19	1				**20**
*P. malariae*	2					**2**
Healthy uninfected persons	108					**108**
**Total**	**130**	**10**	**17**	**28**	**5**	**190**

*Detection level: Band intensity as read independently by two readers.

Microscopic smear examinations served as standard reference.

**Table 4:** Comparing immunochromatographic test strips results and standard method in the screening of clinical samples

	**Positive**	**Negative**	**Total**	Kappa, K*
Positive	59	1	60	**0.9757**
Negative	1	129	130	
**Total**	**60**	**130**	**190**	

*A strong agreement was observed at K= 0.9757 (P<0.005).

## Discussion

Malaria is still one of the world’s most deadly diseases today despite the huge investment in combating the disease. Although *P. falciparum* is the most lethal of all *Plasmodium* species, *P. vivax* is the most widespread and common and thus, responsible for the greatest burden of the disease outside Africa [1,15] and results in close to more than half of the worldwide malaria cases [16]. According to WHO, product testing has shown remarkable improvement in test quality over time, and more high quality tests are being procured over time [17]. Although there is significant improvement in the quality of these assays, only a few that target malaria parasite antigens have really worked to expectation. The major malaria antigens targeted by these RDTs are *P. falciparum* specific HRP-2, pLDH and pan-specific aldolase. The ability of these assays to differentiate the various *Plasmodium* forms is another difficulty. It is, therefore, prudent to develop monoclonal antibodies that can sufficiently differentiate the two most common *Plasmodium* species, *P.vivax* and *P. falciparum*.

The high homology among the *Plasmodium* species confirmed the assertion that many regions in *Plasmodium* aldolase gene are completely or highly conserved [9]. These regions may contribute to determining the authentic common antigenic epitopes among these strains of malaria parasite which can assist in the development of drugs targeting these sites. This common epitopes can be exploited for the development of pan-specific mAbs against *Plasmodium* species. In this study, the cloning and expression of soluble recombinant *P. vivax* specific aldolase antigen and its application in the production of high affinity mAbs for malaria diagnosis is described.

The recombinant antigen was used in immunizing rabbit and mice for the production of polyclonal and monoclonal antibodies respectively. The high titers observed in all immunized animals after the second booster immunization are an indication of the immunogenicity of the recombinant protein. Rabbit anti-PvALDO polyclonal antibodies were used in a novel antibody-capture ELISA for the screening of *P. vivax* specific mAbs. With this screening model, it is possible to screen out very good antibodies that could probably not be detected by the traditional indirect ELISA used by most researchers for hybridoma screening. Three mAbs 14C7, 15F1 and 5H7 were selected after a number of sub-cloning and limiting dilutions. mAb 15F1 had extremely low titer when indirect ELISA was used for antibody screening, but very good titer value with the newly developed antibody-capture method. This means that mAbs with similar characteristics have a greater probability of being rejected when applying the traditional indirect ELISA method of hybridoma screening (Table 1). The reason for this phenomenon is unknown but might probably have been due to overshadowing of the epitopes of the antigen during coating. Selected mAbs were used in the establishment of immunochromatographic test strips for evaluation of assay sensitivity and specificity. All antibodies were of the IgG1 class (Table 2). mAbs 15F1 and 5H7 could favourably pair-up as capture and detection antibodies respectively in immunochromatographic assay for the detection of both recombinant and native aldolase in human blood samples.

*Plasmodium vivax* positive samples (n=60), *P. falciparum* positive samples with no mixed infections (n=20), *P. malariae* samples (n=2) and healthy uninfected blood samples (n=108) were evaluated with the immunochromatographic test strips *versus* microscopic examination. Among the 60 *P. vivax* samples, one false negative was observed for samples with parasite densities < 500 parasites/μl (Table 3). This false negative *P. vivax* sample had a parasitaemia density of 54 parasites/μl. The extremely low level of parasitaemia might have accounted for the inability of the test strip to detect this blood samples. The other 59 *P. vivax* positive samples had parasitaemia density range between 120 to 14,220 parasites/μl. This result indicates that samples with parasitaemia below a detectable range of about 100 parasites/μl are very likely to be undetected. The disparity in the level of sensitivity of the test strip to actively circulating *P. vivax* blood aldolase antigen and its recombinant form might probably be due to the a stronger affinity of these antibodies to the active enzyme. Overall sensitivity and specificity of the immunochromatographic assay were 98.33% (59/60) and 99.23% (129/130), respectively at a 95% CI and Kappa statistics of 0.9757, P<0.005 (Table 4), an indication of a strong agreement between this test and standard methods used in malaria diagnosis. The only observed false positive sample was a *P. falciparum* infected patient that might have been infected with both strains of the parasite. *Plasmodium malariae* samples were also negative. This assay showed high specificity for the *P. vivax* aldolase and not the *P. falciparum* or human blood forms. Because of the scarcity and the difficulty in obtaining the other *Plasmodium* species (*P. ovale, P. knowlesi*), only the *P. vivax, P. falciparum* and *P. malariae* were tested. The high sensitivity and specificity observed in this assay makes it a favourable alternative to the low sensitivities observed in other commercial RDTs in the detection of *P. vivax*[5]. Previous studies have also observed decreased levels of sensitivity in pLDH-specific RTDs for non-*P. falciparum* (*P. vivax*) at parasite densities above 5,000 parasites/μl and higher rate of false negative results in *P. vivax* infections [18,19].

## Conclusion

In conclusion, results from our current evaluation showed that these PvALDO test strips have good specificity and sensitivity for *P. vivax* even at very low parasite densities, it would hence be expedient to further explore these antibodies in a larger population for use as diagnostic tools for rapid and convenient clinical assessment of suspected malaria infections caused by *P. vivax*. An added advantage of this assay would be its ability to adequately differentiate the two most important *Plasmodium* forms (*P. falciparum* and *P. vivax*), a decision that is very critical in the therapeutic treatment of malaria.

## Competing interests

The authors declare that they have no competing interests.

## Authors’ contributions

EED and KK performed the experiments. CN and HW assisted in the protein expression. PW assisted in immunization and hybridoma screening. KK also carried out samples collection and organized the microscopic diagnosis. ST, JW, JW and XW were responsible for the design and application of the study. All authors read and approved the final manuscript.
